# Inhibition of Oxidative Stress and Skin Aging-Related Enzymes by Prenylated Chalcones and Other Flavonoids from *Helichrysum teretifolium*

**DOI:** 10.3390/molecules20047143

**Published:** 2015-04-20

**Authors:** Olugbenga K. Popoola, Jeanine L. Marnewick, Fanie Rautenbach, Farouk Ameer, Emmanuel I. Iwuoha, Ahmed A. Hussein

**Affiliations:** 1 Chemistry Department, University of Western Cape, Private Bag X17, Bellville 7535, South Africa; E-Mails: 3318925@myuwc.ac.za (O.K.P.); Fameer@uwc.ac.za (F.A.); eiwuoha@uwc.ac.za (E.I.I.); 2 Oxidative Stress Research Centre, Institute of Biomedical and Microbial Biotechnology, Faculty of Health and Wellness Sciences, Cape Peninsula University of Technology, P O BOX 1906, Bellville 7535, South Africa; E-Mails: MarnewickJ@cput.ac.za (J.L.M.); rautenbachf@cput.ac.za (F.R.)

**Keywords:** *Helichrysum teretifolium*, anti*-*tyrosinase, anti-elastase, oxidative stress, anti-aging, flavonoids

## Abstract

Ten flavonoid-related structures *viz.* heliteretifolin (**1**), isoxanthohumol (**2**), 2',4',6'-trihydroxy-3'-prenylchalcone (**3**), isoglabranin (**4**), glabranin (**5**), 7-methoxy-isoglabranin (**6**), quercetin (**7**), 4'-methoxyquercetin (**8**), 4'-methoxykaempferol (**9**) and mosloflavone (**10**) were isolated from a *H. teretifolium* methanolic extract and identified. One of them (compound **1**) is reported for the first time from a natural source, while compounds **6**, **8**–**10** were isolated for the first time from the genus *Helichrysum*. The total extract of *H. teretifolium* showed potent antioxidant activity. When tested for total antioxidant capacity compound **3** possesses moderate biological activity compared to **2**, which displayed some of the highest TEAC values (4529.01 ± 2.44; 4170.66 ± 6.72) µM TE/g, respectively. Compounds **7** and **8** demonstrated the highest inhibitory activities on Fe^2+^-induced lipid peroxidation (IC_50_ = 2.931; 6.449 µg/mL); tyrosinase (8.092; 27.573) and elastase (43.342; 86.548). Additionally, the total antioxidant capacities measured as FRAP (4816.31 ± 7.42; 3584.17 ± 0.54) µM AAE/g, and ORAC for hydroxyl radical (7.265 ± 0.71; 6.779 ± 3.40) × 10^6^ and peroxyl radical (17.836 ± 2.90; 12.545 ± 5.07) × 10^3^ µM TE/g were also observed for compounds **7** and **8**, respectively. In conclusion, *H. teretifolium* total extract represents a rich source of bioactive constituents with potent antioxidant and moderate anti-tyrosinase and anti-elastase activities that can help to avert accumulation of free radicals in the body, and could therefore be good candidates for the prevention and/or treatment of skin-related conditions, such as aging. This is the first scientific report on the chemical and biological profile of *H. teretifolium*.

## 1. Introduction

Skin aging is a biological process that induces changes to the structural integrity and physiological function of the skin [1]. Exposure to UV radiation is one of the most significant external stress-inducing factors, and a major cause of premature skin aging. Wrinkle formation is a striking feature of intrinsic and photo-induced skin aging, which are both associated with oxidative stress and inflammatory responses [2]. The aging process is characterized by the progressive loss of structural integrity and physiological changes caused by intrinsic and extrinsic determinants leading to senescence and degradation of biological functions, due to the inability of organisms to adapt to metabolic stress over time [3]. Overaccumulation of free radicals can cause a number of harmful effects in the skin [4] through activation of skin disease-related enzymes, such as tyrosinase and elastase, which can further contribute to skin aging [5]. Oxidative stress occurs when the formation of bioactive oxidation products such as oxidizing agents, free radicals and reactive oxygen species, greatly overwhelms the capacity of the endogenous cellular antioxidant defense system, thus leading to potential damage of the cellular organelles, contributing to the progression of degenerative diseases in humans [6,7]. Tyrosinase is a copper-containing enzyme which catalyzes the first two stages during the process of melanogenesis [8]. Melanin plays a vital role as a photoprotective agent against the harmful effects of UV radiation, and also determines our phenotypic outlook. However, over-accumulation of melanin in specific parts of the skin results in undesirable skin hyperpigmentation [4,9]. Elastase on the other hand, is a proteolytic enzyme involved in the degradation of elastin, leading to skin aging [5].

Plants have long been used in the cosmetic industry, as amongst others as tyrosinase inhibitors which have become increasingly important to prevent hyperpigmentation through the inhibition of enzymatic oxidation. Neutralization of free radicals usually comes from phenolic compounds like flavonoids, so plants rich in phenolics like *Helichrysum* genus can thus contribute.

*Helichrysum teretifolium* (L.) D. Don (Asteraceae) is a straggling subshrub up to 300 mm tall with cream colored bracts, and occasionally tinged pink flowers, widely distributed along the coast of South Africa [10]. Traditionally, many people believe this shrub has magical properties and can be used to protect a house from lighting strikes. To date there is no reports on the plant constituents, however, the genus *Helichrysum* in general is a rich source of phenolic compounds like flavonoids, chalcones and their prenylated derivatives, in addition to the active phloroglucinol phenolics, which biological properties are widely documented. *Helichrysum* are reported to be traditionally used in the treatment of respiratory diseases and in wound dressings, as anti-inflammatory agents and for other skin conditions [11,12].

## 2. Results and Discussion

**Figure 1:**
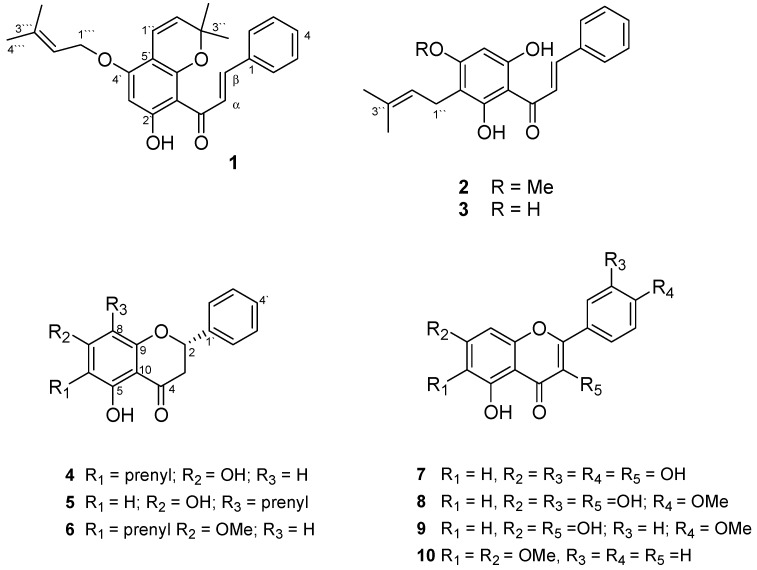
Chemical structures of compounds **1**–**10** isolated from *H. teretifolium*.

Chromatographic purification of a methanol extract of *H. teretifolium* using different techniques including semi-prep HPLC yielded ten pure compounds which included different types of flavonoids. Compound **1** (15 mg) was isolated as an amorphous yellow powder. The HRMS of **1** showed a [M+1]^+^ peak at *m*/*z* 391.1889, corresponding to the molecular formula C_25_H_26_O_4_. The NMR spectra demonstrated chalcone skeleton features with an unsubstituted ring B. ^1^H-NMR showed a singlet at 6.03 (H_3'_), aromatic signals of a monosubstituted phenyl group at 7.58–7.37 (H_2_-H_6_, Table 1); *trans* coupled protons at 8.11, 7.72 (*d* each, *J* = 15.6 Hz), in addition to signals of two prenyl groups, one of them (at C-5') forming a pyran ring with the 6'-OH and containing signals of two *cis* olefinic protons at 6.58, 5.45 (*d*, *J* = 9.6 Hz) and two methyls at 1.52. The other prenyl group forms an ether bond with the 4'-OH and showed signals of a methylene group at 4.53 (2H, *d*, 6.4 Hz), a proton at 5.56 (*t*, *J* = 6.4 Hz) and two methyls at 1.77 s, 1.72 s. The ^13^C-NMR with DEPT-135 confirmed the above data and showed 25 carbons, 15 of them belong to the main chalcone skeleton and the other 10 carbons belonging to the two prenyl groups (Table 1). 2D NMR spectra fully established the structure of **1**, in particular the HMBC correlations spectrum which showed cross peaks between H_1''_/C_5'_, C_6'_, C_4'_; H_1'''_/C_4'_; 5-OH/C_1'_, C_2'_, C_3'_; H_3'_/C_2'_, C_4'_, C_1'_, C_5'_ among others and confirmed the structure as 2'-hydroxy-5',6'-(2,2-dimethylpyrano)-4'-(*O*-prenyl)-chalcone as given in Figure 1. This compound was givedn the trivial name heliteretifoline. To the best of our knowledge and according to the SciFinder database, compound **1** was only described once as synthetic product but has not been isolated before from a natural source [13]. Isoxanthohumol (**2**) and isoglabranin (**4**) were isolated previously from *H. polycladum* [14]. Isoxanthohumol (**2**) was also isolated and identified alongside with 2',4',6'-trihydroxy-3'-prenylchalcone (**3**), and glabranin (**5**) from *H. cymosum* [15,16]. The rare compound **6** was identified previously from *Derris rariflora* [17]. Quercetin (**7**) was isolated form *H. arenarium* [18], while 4′-methoxyquercetin (**8**) was identified from *Dryas octopetala* [19], 4'-methoxykaempferol (**9**) from *Dilleniacea indica* [20], and mosloflavone (**10**) from *Polemonium viscosum* [21]. It is worthy to note the isolation of compounds **6**, **8**–**10** from *Helichrysum* species for the very first time*.*

It is also of interest to note that the NMR of compound **2** showed duplication of some signals (Table 1) because of the free rotation of the single bonds around the carbonyl. This duplication is not observed for the other prenylated chalcones **1** and **3**. 7-Methoxyisoglabranin (**6**) demonstrated very similar NMR spectra to those of isoglabranin (**4**) and glabranin (**5**), however, the distinction between structures **4** and **6** was confirmed by HMBC through the H_1''_ and 5-OH cross peaks with C_5_ and C_6_, while **5** showed the shift of some signals (C_6_ and C_2''_) compared to **4** (Table 1).

**Table 1:** ^1^H- (400 MHz: m, *J* Hz) and ^13^C- (100 MHz) NMR spectral data of isolated compounds **1**–**6** in CDCl_3_.

No.	1		2		3		4		5		6	
	^13^C	^1^H	^13^C	^1^H	^13^C	^1^H	^13^C	^1^H	^13^C	^1^H	^13^C	^1^H
1	135.6		136.6		135.6							
2	128.2	7.58 *dd* , 7.6, 1.6	129.23	7.55 *d* , 6.2	128.3	7.53 *dd* , 1.6, 7.2	79.07	5.37 *dd* , 3.2, 13.2	79.1	5.30 *dd* , 2.5, 13.1	79.3	5.38 *dd* 3.0, 13.2
3	128.9	7.37 *m*	129.88	7.29 *m*	128.6	7.31 *m*	43.44	3.05 *dd* , 13.2, 17.2	43.4	3.05 *dd* , 3.1, 17.2	43.5	3.05 *dd* 13.2, 17.1
								2.79 *dd* , 3.2, 17.2		2.71 *dd* , 13.3, 17.2		2.78 *dd* 3.0, 17.1
4	130.0	7.37 *m*	131.0	7.29 *m*	129.7	7.31 *m*	195.9	-	195.5		195.8	
5	128.9	7.37 *m*	129.9	7.29 *m*	128.6	7.31 *m*	161.2		161.1		161.3	
6	128.2	7.58 *dd* , 7.6, 1.6	129.2	7.55 *d* , 6.2	128.3	7.53 *dd* , 1.6, 7.2	107.0		108.6		110.1	
7							163.8		164.2		165.5	
8							95.5	5.99 *s*	94.8	5.93 *s*	91.0	6.07 *s*
9							161.0		160.6		160.3	-
10							102.9		102.3		102.9	-
1'	106.2	-	106.2		105.2		138.5		138.5		138.5	-
2'	167.3	-	164.7/164.6		163.4		126.1	7.30 *m*	126.1	7.42 *m*	126.1	7.37 *m*
3'	95.5	6.03 *s*	109.2/109.1		106.7		128.8	7.30 *m*	128.7	7.42 *m*	128.9	7.37 *m*
4'	160.6	-	160.9		162.0		128.8	7.30 *m*	128.7	7.42 *m*	128.9	7.37 *m*
5'	103.3	-	91.6	6.05 *s*	94.4	5.80 *s*	128.8	7.30 *m*	128.7	7.42 *m*	128.9	7.37 *m*
6'	155.7	-	164.3/163.9		160.0		126.1		126.1	7.42 *m*	126.1	
α	127.5	8.11 *d* 15.6	128.7/128.8	8.10/8.12 *d* , 15.7	127.9	8.07 *d* , 15.6						
β	142.1	7.72 *d* 15.6	142.8/142.7	7.65 *d* , 15.7	141.7	7.68 *d* , 15.6						
CO	192.8	-	193.8		192.9							
1''	117.0	6.58 *d* , 9.6 Hz	22.0	3.22 *d* , 6.9			21.1	3.33 *d* , 7.2	20.9	3.21 *d* , 6.9	21.0	3.24 *d* , 6.8
2''	124.4	5.45 *d* , 9.6 Hz	124.0	5.09 *t* , 6.9			121.4	5.28 *t* , 7.2	122.1	5.17 *t* , 6.9	122.2	5.16 *tt* , 6.8, 1.4
3''	77.9		136.6				135.7		132.4		131.7	-
4''	27.9	1.52 s	26.0	1.48 *s*			25.8	1.74 *s*	25.6	1.63 *s*	25.8	1.65 *s*
5''	27.9	1.52 s	17.9	1.60 *s*			17.9	1.79 *s*	17.7	1.72 *s*	17.7	1.75 *s*
1'''	65.5	4.53 *d* , 6.4										
2'''	118.8	5.56 *t* , 6.4										
3'''	138.6	-										
4'''	25.8	1.77 *s*										
5'''	18.3	1.72 *s*										
5-OH	-	14.19 *s*		13.61 *s*		13.30 *s*		12.36 *s*				12.03 *s*
7-OMe			56.6	3.70 *s*							55.8	3.81 *s*

Accelerated skin aging is a consequence of direct continuous contact with the environment due to accumulation of reactive oxygen species (ROS). Since aging is becoming a major concern, it is important to focus on its causes and its cure. Although a wide range of factors contribute to skin aging, environmental factors are majorly involved in inducing the stress and enhancing the effect of internal factors in causing aging. Natural antioxidants being cost effective and safer, are the best alternatives for modulating the stress induced by gerontogens. Plant extracts or compounds thus offer new effective treatments to minimize the effects of UV stress and harmful compounds.

Bio-prospecting of natural resources for antioxidants has hence intensified, and a great deal of research is being carried out to identify plants with potent antioxidant activity against skin aging. In this study, we investigated *in vitro* antioxidant capacity of a methanolic extract of *H. teretifolium*. ORAC (perxoxyl and hydroxyl), FRAP, TEAC, and Fe^2+^-induced lipid peroxidation were used as antioxidant capacity and oxidative damage modulation determinants with trolox, ascorbic acid, and EGCG as references.

Compounds **7** and **8** were isolated as active constituents with significant peroxyl (17.836 ± 2.90; 12.545 ± 5.07) × 10^3^ and hydroxyl (7.265 ± 0.71; 6.779 ± 3.40) × 10^6^ µM TE/g radical absorbance capacity and FRAP activity (4816 ± 7.42; 3584.17 ± 0.54) μΜ AAE/g, respectively, with almost the same activity as the commercial antioxidant EGCG (Table 2). The highest ORAC and FRAP values were achieved by **7** due to the presence of 3',4'-dihydroxy group in the B-ring. A further explanation to validate our results was given by Wolfe and Lui, who stated that greater radical stability was due to increased electron delocalization and intramolecular hydrogen bonding between the 3'- and 4'-hydroxyls [22]. Replacement of one of the hydroxyl groups by a methoxyl group (Compound **8**) also contributed to the significant value recorded, possibly due to the presence of the lone pair of electrons on the OMe which can form an intramolecular hydrogen bond with C_3'_-OH. In general, a hydroxyl on the B-ring for the donation of hydrogens to hydroxyl and peroxyl radicals and for transfer of electrons to ferric ion is the most important active group, thereby stabilizing them and giving rise to a relatively stable flavonoid radical. On the other hand, compounds **2** and **3** displayed the highest TEAC value (Table 2). The significance of the hydroxyl configurations in ring-A for such TEAC activity observed for **2** and **3** is less clear. Heim *et al.* gave supporting evidence indicating that 5,7-*m*-di/tri-hydroxy arrangements in ring-A increase TEAC, but such mechanism was not fully explained [23].

**Table 2:** Total antioxidant capacity of *H. teretifolium* constituents.

Sample	Automated Oxygen Radical Absorbance Capacity (ORAC μM TE/g)			FRAP	TEAC
	ROO. (μM/g × 10^3^)	OH. (μM/g × 10^6^)	Prooxidant (μM/g)	μM AAE/g	μM TE/g
HT	1.313 ± 7.54	3.016 ± 5.90	4.163 ± 0.83	511.89 ± 4.61	1179.60 ± 8.20
**1**	2.833 ± 3.88	2.998 ± 1.67	2.036 ± 2.98	ND	ND
**2**	3.113 ± 17.59	2.910 ± 6.00	3.601 ± 2.23	619.91 ± 1.97	4170.66 ± 6.72
**3**	5.025 ± 6.16	3.771 ± 3.02	4.704 ± 0.27	817.94 ± 4.26	4529.01 ± 2.44
**4**	1.063 ± 33.50	2.918 ± 4.13	3.947 ± 0.29	7.052 ± 3.76	43.17 ± 6.26
**5**	0.856 ± 17.35	2.997 ± 0.36	2.971 ± 1.10	67.79 ± 14.27	204.15 ± 2.04
**6**	3.854 ± 5.14	2.955 ± 3.41	3.799 ± 0.60	104.09 ± 4.64	519.25 ± 3.66
**7**	17.836 ± 2.90	7.265 ± 0.71	4.361 ± 0.78	4816.31 ± 7.42	1361.70 ± 1.98
**8**	12.545 ± 5.07	6.779 ± 3.40	8.963 ± 2.79	3584.17 ± 0.54	1009.01 ± 1.98
**9**	10.491 ± 0.97	3.675 ± 1.40	3.790 ± 1.15	191.47 ± 1.39	261.30 ± 4.02
**10**	2.403 ± 2.50	2.909 ± 8.41	6.482 ± 1.55	544.60 ± 6.98	699.66 ± 2.28
EGCG	14.970 ± 5.53	3.911 ± 4.65	6.483 ± 1.19	3326.45 ± 5.76	11545.4 ± 17.28

HT = *H. teretifolium* total extract ND = Not detected; EGCG = Epigallocatechin gallate.

The copper-initiated prooxidant activity, expressed as arbitrary units, was very low (Table 2) when compared with the peroxyl and hydroxyl radical absorbance capacities expressed in 10^3^ and 10^6^, respectively. This result suggests none of the compounds possessed prooxidant activity, possibly due to the non-existence of pyrogallol groups in their respective ring-B substitution patterns.

Oxidative degradation of Lipids is a common consequence of oxidative stress, a process whereby polyunsaturated lipid contents of the biological membrane are susceptible to oxidative damage via their reactions with free radicals, which can lead to lipid peroxidation. Products of lipid peroxidation such as malondialdehyde (MDA), 4-hydroxyl 2-nonenal, and some other alkanals reacts with cell macromolecules to form adducts with significant irreversible effects on cellular functions, and could also promote the aging process. Compounds **7**–**10** (IC_50_ = 2.931; 6.449; 10.520; 10.720 μg/mL, respectively, in Table 3) showed good inhibition of lipid peroxidation. Their significant recorded values are attributable to the presence of an α,β-unsaturated double bond in conjunction with a 4-keto function in their respective structures. Kumar and Pandey [24] established the significance of these features through delocalization of electron on the keto group which resulted to resonance stabilization energy (ring current) in both rings A & B, stabilizing them and giving rise to relatively stable flavonoid radicals formed after the transfer of hydrogen and/or electron. 

**Table 3:** The effects of *H. teretifolium* constituents on inhibition of Fe (II)-induced microsomal lipid peroxidation, tyrosinase and elastase activities.

Sample	Inhibitory Activitis (IC_50_; μg/mL) *		
	Lipid Peroxidation	Tyrosinase	Elastase
HT	16.750	83.517	79.965
**1**	>26.750	>50	>100
**2**	>26.750	>50	>100
**3**	21.276	>50	>100
**4**	>26.750	>50	>100
**5**	>26.750	>50	>100
**6**	23.157	>50	>100
**7**	2.931	8.092	43.342
**8**	6.449	27.573	>86.548
**9**	10.520	29.571	>100
**10**	10.720	38.062	>100
EGCG	0.929	NA	NA
Kojic acid	NA	3.425	NA
Oleanolic acid	NA	NA	9.806

* Data are given as IC_50_ with purified compounds screened at 26.750 μg/mL for inhibition of microsomal lipid peroxidation, while extract (HT) was screened at 100 μg/mL. Anti-tyrosinase activity for both extract and the purified compounds were screened at the effective concentration of 50 μg/mL, while 100 μg/mL was considered as optimum concentration for elastase assay.

Recently, tyrosinase inhibitors have received special attention, due to their alleviating properties that deliver skin lightening and antiaging benefits, caused by undesirable skin hyperpigmentation [25]. The results (Table 3) demonstrated anti-tyrosinase activity in order of **7** > **8** > **9** > **10**. The tyrosinase inhibitory activity of flavones could be ascribed to their ability to chelate copper in the enzyme [8]. Since the partial structure (3,5-dihydroxy-4-keto moiety) which is responsible for the ability to form chelation can be found in our isolated compounds **7**–**10**, it appears very likely that the copper chelation is the main inhibition mechanism of action of flavones as long as their 3, 5-dihydroxyl groups are free [24]. In addition to the above features exhibited by flavones, our results in Table 3 further showed that **7** bearing catechol group at the B-ring was the most effective inhibitor of tyrosinase, lipid peroxidation and elastase (though weak but significant at IC_50_ = 43.342 µg/mL). Other compounds (**8**–**10**), not bearing a catechol group, were not significantly active under the condition assessed. Compound **7** exhibited potent anti-tyrosinase inhibitory activity as shown in Table 3 in accordance with reported data [23,26,27].

## 3. Experimental Section

### 3.1. General Information

Kojic acid, oleanolic acid, epigallocatechin gallate (EGCG), quercetin, 6-hydroxy-2,5,7,8-tetra-methylchroman-2-carboxylic acid (Trolox), 2,2'-azino-bis(3-ethylbenzothiazoline-6-sulfonic acid) diammonium salt (ABTS), potassium peroxodisulphate, fluorescein sodium salt, 2,2'-azobis(2-methyl-propionamidine) dihydrochloride (AAPH), perchloric acid, 2,4,6-tri[2-pyridyl]-s-triazine (TPTZ), iron(III) chloride hexahydrate, Sepharose (wet bead diameter, 60–200 µm), copper sulphate, hydrogen peroxide and N-succyl-(Ala)-3-nitroanilide (SANA), skin enzymes such as tyrosinase (from mushroom), and elastase (from porcine pancreas) were secured from Sigma-Aldrich, Inc. (St. Louis, MO, USA). Organic solvents, methanol, acetonitrile (HPLC grade), ethanol, ethyl acetate, dichloromethane and hexane were supplied by Merck (Cape Town, South Africa). TLC was conducted on normal-phase (Merck) Silica gel 60 PF_254_ pre-coated aluminum plates. Column chromatography was performed using silica gel 60 H (0.040–0.063 mm particle size, Merck) and Sephadex LH-20 (Sigma-Aldrich, Cape Town, South Africa). NMR spectra were recorded on an Avance 400 MHz NMR spectrometer (Bruker, Rheinstetten, Germany) in deuterated chloroform and acetone, using the solvent signals as internal reference. HRMS analysis was conducted on an Ultimate 3000 LC (Dionex Sunnyvale, CA, USA) coupled to a Bruker QTOF with electrospray ionization (ESI) interface working in the positive ion mode.

### 3.2. Preparation of Plant Extracts

The plant material was collected in October 2012 from Jonkershoek Nature Reserve, Western Cape, South Africa. A voucher speciemen was identified by Weitz Franz (Biodiversity Department, UWC) and has been deposited at the Herbarium of the Department of Biodiversity & Plant Biology, University of the Western Cape, Bellville, South Africa with herbarium number Hussein 22/7.

### 3.3. Extraction and Purification of Chemical Constituents

The dried aerial parts (450 g) were blended and extracted with methanol (3.0 L × 2) at room temperature (25 °C) for 48 h. The methanol extract was evaporated till dryness with a rotary evaporator at 45 °C to yield 16 g of residue. The total extract was applied to a silica gel column and eluted using a gradient of hexane (Hex) and ethyl acetate (EtOAc) in the following order of increasing polarity: 100% hex, hex–EtOAc (9:1), (4:1), (7:3), (3:2), (1:1), (2:3), 1:4), (1:9) and 100% EtOAc. The collected fractions (250 mL each) were combined according to their TLC profiles to yield 20 main fractions labeled I–XX.

Main fraction XVIII (1.4 g) was chromatographed on silica gel using a hex/EtOAc gradient (9:1; 7:3; and 100%), then Sephadex (using 95% aqueous ethanol) to produce 4`-methoxykaempferol (**9**, 32 mg, 0.0071%). Fraction III (466 mg) was chromatographed on silica gel using a hex/EtOAc (9:1), then HPLC (90:10 to 100% ACN in 40 min) producing isoglabranin (**4**, R_t_ 15 min, 19 mg, 0.0042%), and heliteretifolin (**1**, R_t_ 23 min, 15 mg, 0.0033%). Fraction X (450 mg) was chromatographed on Sephadex using 5% aqueous ethanol, then HPLC using gradient solvent system of ACN and water (70:30 to 90% ACN in 20 min, then 100% for 20 min) producing glabranin (**5**, R_t_ 27 min, 15 mg, 0.0033%), and 7-methoxyisoglabranin (**6**, R_t_ 32 min, 17 mg, 0.0038%). Fraction XII (505 mg) was chromatographed under the same condition producing mosloflavone (**10**, R_t_ 16 min, 13 mg, 0.0028%), and isoxanthohumol (**2**, R_t_ 30.5 min, 14.8 mg, 0.0033%). Fraction XV (170 mg) was chromatographed on Sephadex using 10% aqueous ethanol, then HPLC using a gradient of ACN and water (40:60 to 50% ACN in 5 min, then 70% for 20 min, followed by 80% for 5 min, and 100% for 10 min), producing quercetin (**7**, R_t_ 21 min, 24 mg, 0.0053%), and 4'-methoxyquercetin (**8**, R_t_ 26.5 min, 19 mg, 0.0042%). Fraction XX (270 mg) was chromatographed on Sephadex using 10% aqueous ethanol, then HPLC using a gradient of ACN and water (90:10 to 100% ACN in 40 min), producing 2',4',6'-trihydroxy-3'-prenylchalcone (**3**, R_t_ 17 min, 21 mg, 0.0047%). The purity of the isolated compounds was monitored by TLC and NMR.

### 3.4. Antioxidant Assays

#### 3.4.1. Ferric-Ion Reducing Antioxidant Power Assay (FRAP)

Working FRAP reagent was prepared in accordance to the methods described previously [28,29]. Absorbance was measured at 593 nm. l-Ascorbic acid was used as a standard and the results were expressed as μM ascorbic acid equivalents per milligram dry weight (μM AAE/g DW) of the test samples.

#### 3.4.2. Automated Oxygen Radicals Absorbance Capacity (ORAC) Assay

ORAC was measured according to the method described by Prior [30], with some modifications [31]. The method measures the antioxidant scavenging capacity of thermal decomposition generated by (a) peroxyl radical of 2,2'-azobis (2-aminopropane) dihydrochloride (AAPH; ORAC_ROO_**^.^** assay), (b) hydroxyl radical (ORAC_OH_**^.^** assay), generated by H_2_O_2_-Cu^2+^ (H_2_O_2_, 0.3%; Cu^2+^ [as CuSO_4_], 18 µM, or (c) Cu^2+^ [as CuSO_4_], 18 µM as a transition metal oxidant at 37 °C. ORAC values were expressed as micromoles of Trolox equivalents (TE) per milligram of test sample, except when Cu^2+^ (without H_2_O_2_) was used as an oxidant in the assay. In the presence of Cu^2+^ without H_2_O_2_, test samples acted as prooxidants rather than antioxidants in the ORAC assay. The copper-initiated prooxidant activity was calculated using [(Area_Blank_ − Area_Sample_)/Area_Blank_] × 100 and expressed as prooxidant units; one unit equals the prooxidant activity that reduces the area under the fluorescein decay curve by 1% in the ORAC assay.

#### 3.4.3. Trolox Equivalent Absorbance Capacity (TEAC) Assay

The total antioxidant activity of test samples were measured using previously described methods [32,33]. Absorbance was read at 734 nm at 25 °C in a plate reader and the results were expressed as μM Trolox equivalents per milligram dry weight (μM TE/g DW) of the test samples.

#### 3.4.4. Inhibition of Fe (II)-Induced Microsomal Lipid Peroxidation Assay

Rat liver microsomes were isolated from S9 rats using sepharose column with 0.01 M potassium phosphate buffer; pH 7.4, supplemented with 1.15% KCl at 5 °C. A modified assay described by Snijman *et al.* [34] with little modifications. Absorbance was measured at 532 nm and the percentage inhibition of TBARS formation relative to the positive control was recorded.

#### 3.4.5. Tyrosinase Enzyme Assay

This assay was performed using the method described of Chompo *et al.* and Vardhan *et al.* [4,35] with slight modifications. Samples were dissolved in DMSO to a stock solution of 1 mg/mL, and further dilutions were then done with 50 mM sodium phosphate buffer (pH 6.5) for all working solutions. Kojic acid was used as control drug. In the wells of a 96-well plate, 70 µL of each sample working solution was combined with 30 µL of tyrosinase (500 Units/mL in sodium phosphate buffer) in triplicate. After incubation at room temperature for 5 min, 110 µL of substrate (2mM l-Tyrosine) was added to each well. Final concentrations of the crude extract, isolated compounds, and positive control ranged from 1.0–100 µg/mL. Incubation commenced for 30 min at room temperature and the enzyme activity was determined by measuring the absorbance at 490 nm. The percentage of tyrosinase inhibition was calculated as follows:

Tyrosinase inhibition (%) = [(A − B) − (C − D)]/(A − B) × 100(1)

where A is the absorbance of the control with the enzyme, B is the absorbance of the control without the enzyme, C is the absorbance of the test sample with the enzyme and D is the absorbance of the test sample without the enzyme.

#### 3.4.6. Elastase Inhibition Assay

Inhibition of elastase by the test samples was assayed using N-succyl-(Ala)3-nitroanilide (SANA) as the substrate, monitoring the release of *p*-nitroanilide by the method described by Chompo *et al.* [4] with little adjustment. The inhibitory activity determined the intensity of colour released during cleavage of SANA by the action of elastase. Briefly, 1 mM SANA was prepared in 0.1 M Tris-HCl buffer pH 8.0 and 200 µL of this solution was added to the 20 µL of sample solution in a 96-Well plate. The mixtures were vortexed and preincubated for 10 min at 25 °C and then 20 µL of elastase from porcine pancrease (0.03 units/mL) was added. The mixtures were further incubated for 10 min and the absorbance was measured at 410 nm. Methanol was used as control, while oleanolic acid used as a positive control. The percentage of elastase inhibition was calculated as follows:

Elastase inhibition (%) = (1 − B/A) × 100

where A is the enzyme activity without sample and B is the activity in the presence of the sample.

## 4. Conclusions

The numbering, positioning and substitution patterns displayed by the phenyl rings A and B in the isolated compounds resulted in different biological activities. It may be collectively stated that most of the *H. teretifolium* constituents display efficient hydroxyl and peroxyl radical absorbance capacities, inhibiting lipid peroxidation, as well as serving as good sources with anti-tyrosinase activity in *in vitro* systems. This could be attributed to the good quality flavonoids it contained which may also contribute to its biomedical applications. The present work is the first scientific report on *H. teretifolium* and the results suggest that the extract of this plant or its individual constituents might become natural agents to inhibit oxidative stress and tyrosinase, both playing an important role in skin aging.
